# Pre-transplant kidney quality evaluation using photoacoustic imaging during normothermic machine perfusion

**DOI:** 10.1016/j.pacs.2024.100596

**Published:** 2024-02-09

**Authors:** Anton V. Nikolaev, Yitian Fang, Jeroen Essers, Kranthi M. Panth, Gisela Ambagtsheer, Marian C. Clahsen-van Groningen, Robert C. Minnee, Gijs van Soest, Ron W.F. de Bruin

**Affiliations:** a Erasmus MC, Cardiovascular Institute, Thorax Center, Department of Cardiology, Dr. Molewaterplein 40, 3015 GD, Rotterdam, the Netherlands; b Erasmus MC Transplant Institute, Department of Surgery, Division of HPB and Transplant Surgery, Erasmus Medical Center, Rotterdam, Dr. Molewaterplein 40, 3015 GD, Rotterdam, the Netherlands; c Department of Molecular Genetics, Erasmus Medical Center, Dr. Molewaterplein 40, 3015 GD, Rotterdam, the Netherlands; d Department of Pathology and Clinical Bioinformatics, Erasmus Medical Center, Dr. Molewaterplein 40, 3015 GD, Rotterdam, the Netherlands; e Department of Precision and Microsystems Engineering, Faculty of Mechanical Engineering, Delft University of Technology, Van Mourilk Broekmanweg 6, 2628 XE, Delft, the Netherlands; f Wellman Center for Photomedicine, Massachusetts General Hospital, 40 Blossom Street, Boston, MA 02114, USA; g Department of Radiotherapy, Erasmus Medical Center, Dr. Molewaterplein 40, 3015 GD, Rotterdam, the Netherlands; h Department of Vascular Surgery, Erasmus Medical Center, Dr. Molewaterplein 40, 3015 GD, Rotterdam, the Netherlands

**Keywords:** Photoacoustics, Kidney, Transplantation, Normothermic machine perfusion, Oxygenation, Oxygen saturation, Pre-transplant kidney quality

## Abstract

Due to the shortage of kidneys donated for transplantation, surgeons are forced to use the organs with an elevated risk of poor function or even failure. Although the existing methods for pre-transplant quality evaluation have been validated over decades in population cohort studies across the world, new methods are needed as long as delayed graft function or failure in a kidney transplant occurs. In this study, we explored the potential of utilizing photoacoustic (PA) imaging during normothermic machine perfusion (NMP) as a means of evaluating kidney quality. We closely monitored twenty-two porcine kidneys using 3D PA imaging during a two-hour NMP session. Based on biochemical analyses of perfusate and produced urine, the kidneys were categorized into ‘non-functional’ and ‘functional’ groups. Our primary focus was to quantify oxygenation (*sO*_*2*_) within the kidney cortical layer of depths 2 mm, 4 mm, and 6 mm using two-wavelength PA imaging. Next, receiver operating characteristic (ROC) analysis was performed to determine an optimal cortical layer depth and time point for the quantification of *sO*_*2*_ to discriminate between functional and non-functional organs. Finally, for each depth, we assessed the correlation between *sO*_*2*_ and creatinine clearance (*CrCl*), oxygen consumption (*VO*_*2*_), and renal blood flow (RBF).

We found that hypoxia of the renal cortex is associated with poor renal function. In addition, the determination of *sO*_*2*_ within the 2 mm depth of the renal cortex after 30 min of NMP effectively distinguishes between functional and non-functional kidneys. The non-functional kidneys can be detected with the sensitivity and specificity of 80% and 85% respectively, using the cut-off point of *sO*_*2*_ < 39%. Oxygenation significantly correlates with RBF and *VO*_*2*_ in all kidneys. In functional kidneys, *sO*_*2*_ correlated with *CrCl,* which is not the case for non-functional kidneys.

We conclude that the presented technique has a high potential for supporting organ selection for kidney transplantation.

## 1 Introduction

Kidney transplantation is considered an optimal treatment for end-stage renal disease (ESRD) [1]. Due to the lack of available kidney donations, patients have to wait up to 10 years for a suitable organ depending on geographical location. Consequently, many patients die on the waiting list [2], [3], [4], [5], [6], [7], [8].

Donations from living donors or otherwise healthy donations after brain death (DBD) are preferable for transplantation. Nevertheless, to increase the donor pool, the use of kidneys from expanded criteria donors (ECD), i.e*.*, older individuals including those with circulatory conditions, and donation after circulatory death (DCD) donors have become more prevalent [9], [10]. The use of ECD and DCD kidneys is associated with an elevated risk of impaired graft function and even failure in a kidney transplant [9], [11]. Besides, the quality of a donated kidney is affected by multiple parameters which include donor characteristics, organ procurement, preservation, transplantation, and post-transplant management [12], [13]. Therefore, successfully expanding the donor pool with ECD and DCD donors requires a rigorous and objective assessment of kidney function to mitigate severe risks.

Currently, pre-transplant kidney quality assessment predominantly relies on donor characteristics, kidney macroscopic appearance, and renal biopsies. However, these approaches have several limitations. Evaluations based on donor characteristics and macroscopic appearance are subjective and vary among different transplant centers and even individual clinicians. The Kidney Donor Profile Index (KDPI) is an internationally accepted metric that relies on the donor’s characteristics [13], [14]. However, the number of medical centers that have integrated KDPI into their practice is limited. Renal biopsy can provide valuable insights into the histological features of kidney transplants but carries associated risks due to its invasive nature. Besides, histological examination is subject to sampling variability as it provides highly localized information. Biopsies overestimate organ damage and thus do not reliably predict the performance of the transplant [14], [15], [16]. Finally, the histological assessment is not a real-time measure and the kidney has to be preserved until the outcome is known, which is undesirable [17].

Alternatively, medical imaging can provide valuable functional and morphological information about the kidney [18]. Ultrasound (US) is a first-choice modality because of its usability, safety, and relatively low price, but is limited by aberration in obese patients. Doppler US methods can be used to characterize flow and perfusion in the transplanted organ [19]. X-ray computed tomography (CT) can provide high-resolution imaging and also enables CT angiography at low cost, but comes with the downside of ionizing radiation exposure [20]. Magnetic resonance imaging (MRI) is not preferred because of limited access and relatively high cost [21]. More experimental approaches include fluorescence angiography with indocyanine green (ICG) dye to measure the perfusion of a kidney cortex [22] up to 1 cm depth [23]. Photoacoustic (PA) imaging has been applied to detect fibrosis in ex-vivo mouse and pig kidneys [24]. Our work further explores an application of PA for pre-transplant functional kidney quality evaluation and aims to investigate the clinical value of a combination of PA and normothermic machine perfusion (NMP).

Photoacoustic imaging is a non-invasive and non-ionizing modality, which can provide real-time imaging data with molecular specificity relying on spectroscopic contrast. Photoacoustics capitalizes on the acoustic wave that is emitted by chromophores (i.e., light-absorbing molecules) after being exposed to a short light pulse. Unlike optical imaging modalities, PA can image up to a depth of a few centimeters, making it suitable for imaging renal cortex. The unique absorption spectrum of each chromophore enables its quantification within the sample [25], [26].

We hypothesize that, since PA imaging can provide real-time and objective information about the molecular composition of the kidney, it can potentially improve the quality assessment in terms of speed and accuracy of pre-transplant kidney quality evaluation.

Normothermic machine perfusion is an experimental technique that aims to retain or improve and assess kidney function where the organ is supplied with perfusate enriched with nutrients and oxygen carriers at body temperature to mimic the physiological environment of a human body [17], [27]. Also, NMP can be utilized as a platform for testing kidney viability, such as assessed by oxygen consumption, which we exploit in our study [28]. The macroscopic appearance, renal blood flow (RBF), and urine output are the most intuitive indicators to assess kidney quality, but they vary greatly based on the different perfusate components and kidney size [29], [30]. More specifically, biomarkers such as glomerular filtration rate and oxygen consumption can be measured by perfusate and urine samples. These biomarkers reflect renal function and viability. However, the analysis requires time-consuming laboratory procedures, and waste products of metabolism such as creatinine need to be added to the perfusate to analyze the filtration rate.

Several studies have explored the applications of medical imaging of kidneys during NMP for pre-transplant kidney assessment. Markgraf et. al. applied hyperspectral optical imaging on the cortical area of a kidney during NMP, which allows the quantification of oxygen saturation or water content on the organ's surface [31] [32]. Alternatively, Schuller et al. used MRI to assess the renal flow distribution of a kidney during NMP [33]. Finally, Fang et. al demonstrated that laser speckle contrast imaging (LSCI) can visualize cortical perfusion which correlates with oxygen consumption and urine production [34].

The goal of our work is to investigate the value of PA imaging during NMP as a tool for kidney quality evaluation before transplantation which is also the novelty of the presented study. We investigate whether *sO*_*2*_ measured with PA is associated with organ quality. We also investigate how oxygenation correlates with organ quality assessed by biochemical metrics, such as creatinine clearance (*CrCl*), oxygen consumption (*VO*_*2*_), and by functional metrics such as renal blood flow (RBF).

## 2 Materials and methods

### 2.1 Experimental setup design

**Fig. 1:**
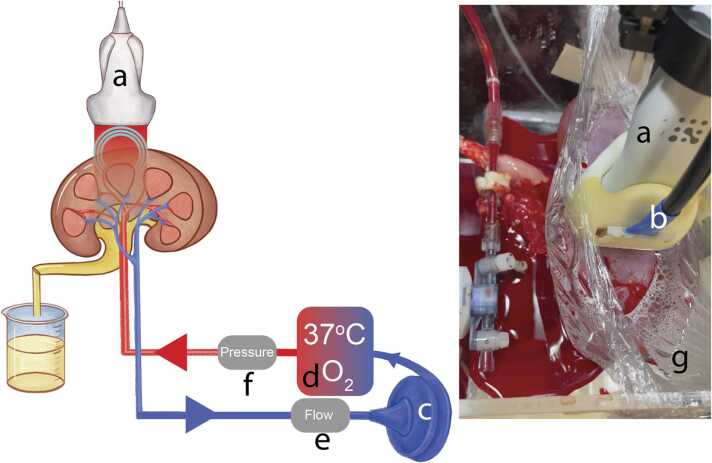
The experimental PA NMP setup. (a) US transducer equipped with (b) laser fiber bundles. (c) Centrifugal pump head. (d) Oxygenator with a heat exchanger. (e) Flow sensor. (f) Pressure sensor. (g) Interface film filled with water.

The schematic of the experimental setup is depicted in Fig. 1. The setup is comprised of a custom-made NMP setup and a Vevo3100 LAZR-X system (Fujifilm VisualSonics, Toronto, Canada) equipped with an MX201 ultrasound (US) linear transducer array (VisualSonics, Toronto, Canada) operating at 15 MHz central frequency. The transducer is attached to a linear translational stage enabling 3D image acquisition.

The NMP system consists of a centrifugal pump head (BPX-80 Bio-Pump™ Plus, Medtronic, Minneapolis, MN, USA) equipped with a pump drive (BVP-BP, Harvard Apparatus, Germany), an oxygenator with a heat exchanger (Hilite 1000, Medos, Germany), and a thermocirculator (E100, Lauda, Germany). A flow probe (73–4755, Harvard Apparatus, Germany) is connected in-line with the kidney, and a pressure sensor (APT300, Harvard Apparatus, Germany) is directly connected to the renal artery cannula (Organ Recovery Systems, Itasca, USA). The setup is controlled by a commercially available electronic controller (PLUGSYS Servo Controller for Perfusion, Harvard Apparatus, Germany) which enables both flow and pressure-directed perfusions. The choice of the design was motivated by the reusability of the materials as opposed to commercially available systems.

### 2.2 Kidney preparation and sample collection

Twenty-two porcine kidneys from female landrace pigs were obtained from a local slaughterhouse. The kidneys were explanted by a designated butcher following a standardized slaughter procedure. Once the kidneys were explanted, back-table preparation was performed by 2 of the authors (YF and GA). Since the organs were obtained from pigs intended for meat consumption, no approval from the animal ethics committee was required.

Directly after the animal slaughter, two liters of blood were collected in a bucket containing 25000 IU of heparine (LEO Pharma A/S, Ballerup, Denmark), and filtered by leukocyte filter (BioR 02 plus, Fresenius Kabi, Zeist, the Netherlands). After cannulating the renal arteries with a 5 mm straight cannula (Organ Recovery Systems, Itasca, USA), the kidneys were flushed with 500 mL Ringer’s lactate (Baxter BV, Utrecht, the Netherlands) at a temperature of 4 °C. Warm ischemia time (WIT) was calculated from the blood collection until cold flushing. Next, the kidneys were loaded into LifePort (Organ Recovery Systems, Itasca, USA) devices for transportation and cold preservation until NMP. In the LifePort, the kidney was perfused with 4 °C Belzer UW machine perfusion solution (Belzer MPS, Bridge to Life Ltd., London, UK), at 30 mmHg of pressure.

In this study, cardiac arrest occurred in pigs and kidneys experienced a period of warm ischemia due to the slaughter procedure. This scenario mimics the DCD condition as DCD donors also experience cardiac arrest before organ donation in clinical practice [35]. Fourteen kidneys underwent 30 min WIT, and the remaining 8 kidneys underwent prolonged WIT of 75 min. A kidney from a healthy, young pig subjected to 75 min WIT was proven to be a model for DCD kidney with substantial renal injury which is actively used in research including the Netherlands [36], [37].

After removing the kidneys from the LifePort, they were flushed with 100 mL Ringer’s lactate at 4 °C to remove the remaining UW solution. They were subsequently perfused with autologous blood-based solution (Table S1) at 37 °C with a controlled pressure of 70 mmHg. Carbogen (95% O_2_ and 5% CO_2_) was supplied via the oxygenator at flow rate of 500 mL/min.

During NMP, each kidney was positioned inside a custom-made chamber. To facilitate acoustic coupling and prevent direct contact between the kidney and the transducer, transparent polyethylene film was used to cover the kidney (Fig. 1-g). The transducer was positioned above the kidney and the space between the transducer and the film was filled with water for acoustic coupling. There was no water leakage through the film into the chamber during the measurements.

Before and after NMP, renal cortical biopsies were collected for histological examination. The collected blood was used as perfusate. Renal blood flow was recorded every 10 min. The perfusate samples from renal artery and vein as well as urine samples were collected at 30 min, 60 min, and 120 min of NMP for further laboratory analysis. Urine production was recorded at the same time points. The PA data was acquired approximately every 20 min

### 2.3 Renal function assessment

Arterial and venous blood gas analyses from the perfusate samples were performed to calculate *VO*_*2*_:

(1) $$VO_{2}=\left(\frac{C_{a}O_{2}-C_{v}O_{2}}{m}\right)\times Q$$

where $C_{a}O_{2}$ and $C_{v}O_{2}$ are arterial and venous oxygenation contents (mLO_2_/L), m is kidney mass (gram), and *Q* is RBF (dL/min).[35].

The perfusate and urine concentrations of creatinine were measured using routine clinical assays to calculate *CrCl*:

(2) $$\mathrm{CrCl}=\left(\frac{C_{\mathrm{Cr}\;\mathrm{in}\;\mathrm{Urine}\times Q_{\mathrm{urine}}}}{{C_{\mathrm{Cr}}}_{\;in\;Perfusate}}/m\right)\times 100$$

where *C*_*Cr in Urine*_ is creatinine concentration in urine (mmol/L), *Q*_*Urine*_ is urine output rate (mL/min), *C*_*Cr in Perfusate*_ is creatinine concentration in perfusate (mmol/L) [35].

### 2.4 Pathological assessment

Renal biopsies were fixed in 4% buffered paraformaldehyde for 48 h and then transferred to 70% ethanol until embedded in paraffin. The embedded tissues were cut into 4 µm slices and stained with periodic acid-Schiff (PAS). Acute tubular necrosis (ATN) was evaluated and scored on a semi-quantitative scale of 0 to 3 (0 - no changes, 1 - mild, 2 - moderate, 3 - severe changes) by a renal pathologist (MCvG) blinded to the study. The score was based on the degree of brush border loss, tubular dilatation, epithelial vacuolation, thinning, sloughing, and luminal debris/casts [38], [39].

### 2.5 Kidney classification

We used the values of *VO*_*2*_ and *CrCl* to divide the kidneys into Group 1 and Group 2. Group 1 was referred to as "non-functional" kidneys, whilst Group 2 comprised "functional" kidneys. The criteria for defining these groups were based on the studies that utilized a comparable NMP setup and perfusate. Specifically, non-functional kidneys were defined as exhibiting *CrCl* ≤ 1 mL/min/100 g and *VO*_*2*_ ≤ 2.6 mL/min/100 g at 120 min of NMP, whilst functional kidneys were defined by *CrCl* > 1 mL/min/100 g and *VO*_*2*_ > 2.6 mL/min/100 g [40], [41].

In practice, a kidney transplant showing inferior function is associated with low urine production, low RBF, and high intrarenal resistance (IRR = arterial pressure/RBF) [42], [43]. Therefore, the separation was validated by comparing total urine production, RBF, and IRR between the groups at the end of NMP. The significance of the difference between the groups was calculated with the Wilcoxon test.

### 2.6 Image data acquisition and data analysis

During each measurement, we acquired 3D PA and US B-mode image data by mechanically translating the US transducer above each kidney over a 45.5 mm distance with a step size of 0.5 mm, resulting in 91 acquired imaging frames (20 mm width and 30 mm depth). Each 2D PA image dataset was acquired at two wavelengths, 750 nm and 850 nm with persistence set as low, at each translational step. This pair of wavelengths is used in ‘Oxy Hemo’ mode embedded in the Vevo 3100 LAZR-X system, but also in the LED-based PA Cyberdyne Acoustic X system (Cyberdine Inc., Tsukuba, Japan). Therefore, the use of those wavelengths can simplify the clinical translation of the proposed technique. Interleaved acquisition was employed to collect the PA and B-mode 2D image data. Although we ensured the kidney was stable during the measurement, small accident motions were observed in B-mode images during the acquisition procedure. Those motions were neglected due to its low amplitude and persistence set.

The beamformed PA data at both wavelengths was exported from the Vevo3100 LAZR-X system for further processing. The data was utilized to calculate the relative concentrations of oxygenated and non-oxygenated hemoglobin $C_{\mathrm{HbO}2}$ and $C_{\mathrm{HbO}2}$:

(3) $$\left(\begin{array}{c} C_{\mathrm{Hb}}\\ C_{\mathrm{HbO}2} \end{array}\right)={\left(\begin{array}{c} \mu_{\mathrm{Hb}}\left(750\mathrm{nm}\right)\;\mu_{\mathrm{HbO}2}\left(750\mathrm{nm}\right)\\ \mu_{\mathrm{Hb}}\left(850\mathrm{nm}\right)\;\mu_{\mathrm{HbO}2}\left(850\mathrm{nm}\right) \end{array}\right)}^{-1}\left(\begin{array}{c} p\left(750\mathrm{nm}\right)\\ p\left(850\mathrm{nm}\right) \end{array}\right),$$

where p is an acquired PA pressure, $\mu_{\mathrm{HbO}2}$ and $\mu_{\mathrm{Hb}}$ are absorption spectra of oxygenated and non-oxygenated hemoglobin taken from [10].

The acquired PA data was utilized to calculate the oxygenation $sO_{2}$ (%):

(4) $$sO_{2}=\frac{C_{\mathrm{HbO}2}}{C_{\mathrm{Hb}}+C_{\mathrm{HbO}2}}\times 100\%.$$

Fluence compensation was not applied to the PA data because it requires prior knowledge of the optical properties of the light propagation media. Even if those properties can be estimated for the optically heterogeneous renal cortex [44], [45], the cellular changes induced in the kidneys by warm ischemia can alter those parameters and result in inaccurate compensation. The time gain compensation (TGC) was manually adjusted to enhance the PA signal within the PA image and saved in a preset. We applied this preset to measure all kidneys.

### 2.7 Statistical analysis

For the analysis, we calculated *sO*_*2*_ for each slice within the renal cortical layer of depths 2, 4, and 6 mm, respectively. The cortex surface was segmented from the B-mode US data. To determine the optimal settings for measurement of *sO*_*2*_, we compared the *sO*_*2*_ values at 30 min, 60 min, and 110 min of NMP. The exact *sO*_*2*_ values for the time points were obtained by linear interpolation of the initially acquired data. The significance of the difference in *sO*_*2*_ between Group 1 and Group 2 was calculated using the Wilcoxon test. We also applied the Kruskal-Wallis test to statistically compare the *sO*_*2*_ values at different depths at each time point. Finally, we performed receiver operating characteristic (ROC) analysis to determine an optimal depth and time point for measurement.

We examined the correlation between *sO*_*2*_ and the RBF, *CrCl*, and *VO*_*2*_ over time in Group 1 and Group 2 using the Spearman linear correlation test. We represented RBF, urine production, and *sO*_*2*_ data in box plots (median, lower, and upper quartiles, whiskers indicate the maximum and minimum data point unless the data point is an outlier) [46]. All image and data processing was performed in Matlab 2023a (The MathWorks, Natick, MA, USA).

## 3 Results

### 3.1 Kidney group division

**Fig. 2:**
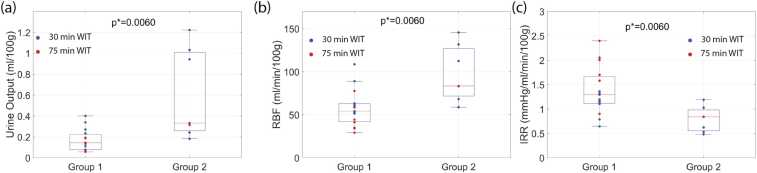
(a) Total urine production measured in Group 1 and Group 2 at 120 min of NMP. (b) RBF measured in Group 1 and Group 2 at 120 min of NMP. (c) IRR measured in Group 1 and Group 2 at 120 min of NMP. The Blue and Red dots represent kidneys subjected to 30 min and 75 min WIT respectively.

According to the criteria defined in Section 2.4, the kidneys were divided into two groups: Group 1 (non-functional, n = 15) and Group 2 (functional, n = 7). There were significant differences in the tolerance to warm ischemia among different individual kidneys. Eight kidneys with 30 min WIT and 7 kidneys with 75 min WIT were included in Group 1, while Group 2 consisted of the remaining kidneys with 30 min WIT and one kidney with 75 min WIT. The RBF, IRR metrics, and total urine production at the end of NMP for both groups are shown in Fig. 2. The statistically significant difference between the groups in RBF and urine production justifies the rationale behind the group division.

### 3.2 *sO*_*2*_ measurement

**Fig. 3–:**
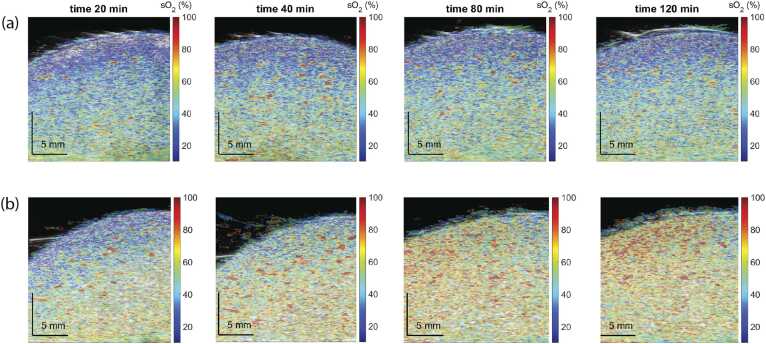
Oxygenation sO_2_ map measured at 20 min, 40 min, 80 min, and 120 min of NMP for a kidney from (a) Group 1 and (b) Group 2. The mentioned time points are approximate since the measurement at exact time points was impossible due to practical reasons.

**Fig. 4:**
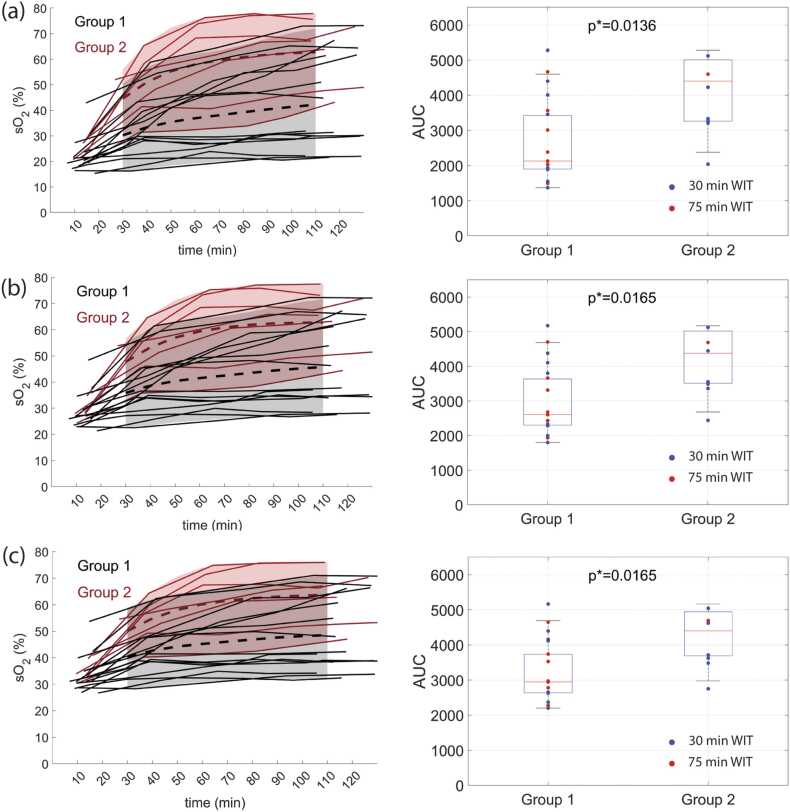
(left) Oxygenation over time and (right) AUC per kidney for the organs from (black) Group 1 and (red) Group 2 measured within the renal cortical layer of depths (a) 2 mm, (b) 4 mm, and (c) 6 mm. The dashed black and red lines depict the mean sO_2_ levels for kidneys from Group 1 and Group 2 respectively. The gray and light red areas depict 90% confidence interval for kidneys from Group 1 and Group 2 respectively. The Blue and Red dots represent kidneys subjected to 30 min and 75 min WIT respectively.

The *sO*_*2*_ maps over time in kidneys from Group 1 and Group 2 are presented in Fig. 3, where an increase of *sO*_*2*_ in the kidney of Group 2 over time can be observed. The mean *sO*_*2*_ values in the cortical layers of 2 mm, 4 mm, and 6 mm over time are shown in Fig. 4. Overall, the kidneys from Group 2 express higher *sO*_*2*_ over time. The area under the curve (AUC) measured in Group 1 is significantly lower than that in Group 2. The significance is the highest for *sO*_*2*_ measured in the renal cortex of 2 mm depth.

**Fig. 5:**
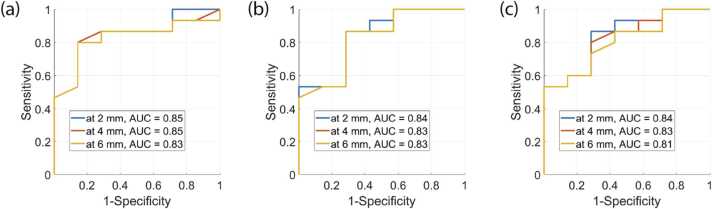
ROC curves for sO_2_ as a classifier between the kidneys from Group 1 and Group 2 at (a) 30 min, (b) 60 min, and (c) 110 min after NMP start measured within the renal cortical layer of depths 2 mm, 4 mm, and 6 mm.

The results presented in Fig. 4 suggest that the most significant difference between *sO*_*2*_ in Group 1 and Grpoup 2 can be reached by measuring *sO*_*2*_ within 2 mm of cortical depth, and the contrast diminishes with increasing layer depth. The Kruskal-Wallis test did not reveal a statistically significant difference between *sO*_*2*_ measured at the same time points but at different depths, except in Group 1 at 30 min of NMP (p = 0.01). The ROC analysis shown in Fig. 5 confirms this result: the largest AUC = 0.85 was calculated from ROCs derived from *sO*_*2*_ measurements measured in 2 mm and 4 mm layer thickness after 30 min of NMP.

**Fig. 6:**
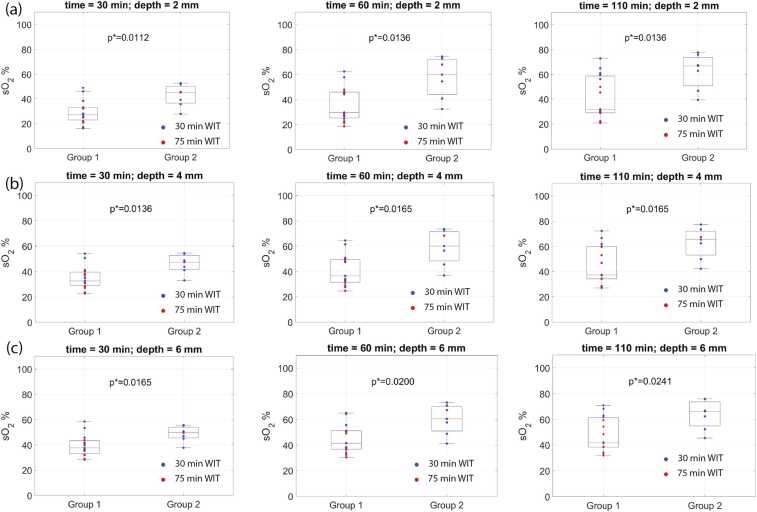
Oxygenation measured within the renal cortical layer of (a) 2 mm, (b) 4 mm, (c) 6 mm depth at time points 30 min, 60 min, and 110 min from the start of NMP. Red and Blue markers depict the kidney samples subject to 75 min and 30 min WIT respectively.

By measuring *sO*_*2*_ within 2 mm cortical depth after 30 min of NMP, the kidneys from Group 2 can be discriminated by thresholding the *sO*_*2*_ value below 39% resulting in sensitivity and specificity of 80% and 86% respectively. By measuring *sO*_*2*_ within 2 mm cortical depth after 60 min of NMP, the kidneys from Group 2 can be discriminated by thresholding the *sO*_*2*_ value below 54% resulting in sensitivity and specificity of 71% and 86% respectively (Fig. 5 and Fig. 6).

**Fig. 7:**
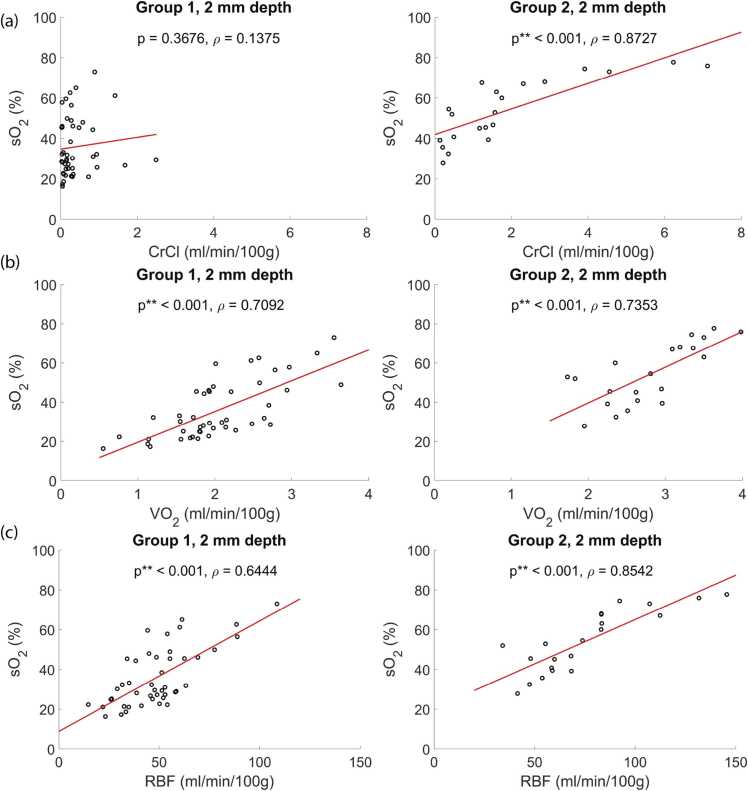
Correlation between sO_2_ and (a) CrCl, (b) VO_2,_ and (c) RBF measured at 2 mm depth in Group1 and Group2. The analysis utilized the data acquired at all-time points.

The correlation plot between *sO*_*2*_ measured at 2 mm depth and *CrCl*, *VO*_*2*_, and RFB are depicted in Fig. 7. In Group 1, *sO*_*2*_ correlates with RBF, and *VO*_*2*_, whilst it does not correlate with *CrCl*. In Group 2, *sO*_*2*_ correlates positively with *CrCl*, RBF, and *VO*_*2*_.

### 3.3 Visual and pathological inspections

**Fig. 8:**
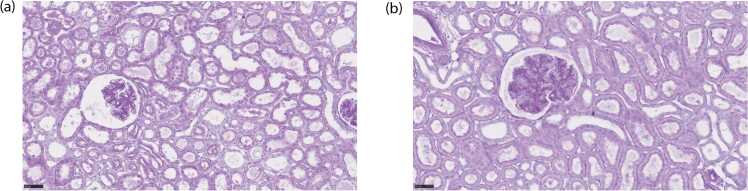
Representative histological images of biopsies taken from kidneys from (a) Group 1 (grade 3) and (b) Group 2 (grade 2) after 120 min of NMP. Moderate severity of acute tubular injury, tubular dilation, and tubule brush loss can be observed in both cases.

Visual assessment of the kidneys before NMP did not reveal any substantial differences in organ appearance. Additionally, during the perfusion, the kidneys had a similar pink appearance. The representative histological images of biopsies are shown in Fig. 8, and the full overview of the histology grades is provided in supplementary Table S2. After NMP, 5 kidneys from Groups 1 and 2 kidneys from Group 2 received grade 3, revealing severe ATN, whilst the rest of the kidneys received grade 2, revealing moderate ATN.

For the kidneys with grade 2, the pathological examination revealed partial loss of brush border in tubular epithelial cells, slight enlargement of Bowman’s space in glomeruli, moderate tubular dilation, interstitial edema, and the presence of tubular casts in renal tubules. These changes indicate moderate acute ischemic injury. Kidneys with grade 3 exhibited more pronounced alterations, including more significant brush border loss, marked enlargement of Bowman’s space, and higher prevalence of cast formation, indicating severe acute ischemic injury.

## 4 Discussion

In this study, we explored the value of PA imaging for kidney evaluation before transplantation. Specifically, we investigated *sO*_*2*_ within the renal cortex during NMP as a quality metric to indicate the suitability of kidneys for transplantation.

The novelty of our work is that we performed PA imaging while the organ was connected to NMP. This technique can help a transplant surgeon to make a better decision about the translatability of a donated kidney. The main contribution of this work is that we demonstrated, through ex-vivo experiments, the association between hypoxia in the renal cortex and low levels of *CrCl* and *VO*_*2*_. We hypothesize that the relatively low level of cortical *sO*_*2*_ observed during NMP is associated with inferior function. This hypothesis is further supported by the findings of low urine production, low RBF, and high IRR in these kidneys.

Hypoxia is a consequence of warm ischemia, where proximal tubular cells swell, resulting in increased IRR and decreased flow through the renal cortex [47]. Consequently, prolonged warm ischemia disrupts the oxygen supply, leading to significant hypoxia which can be detected by PA imaging. There is an overlap in *sO*_*2*_ values between Group 1 and Group 2 despite the statistically significant difference between the groups at all time points. Low *sO*_*2*_ in a renal cortex indicates a high risk of inferior renal function and transplant failure, while high *sO*_*2*_ does not necessarily guarantee proper renal function and needs to be interpreted cautiously together with other functional markers.

Our results suggest that the best time point to measure *sO*_*2*_ is after 30 min of NMP within 2 mm of the renal cortical depth. This can be explained by the fact that in the first 30 min of NMP, substantial shifts occur in various indicators of renal function and viability due to the abrupt transition from hypothermic to normothermic preservation. The sensitivity of these indicators to even minor differences in measurement time introduces variability, diminishing the robustness of the results. Therefore, we suggest that the optimal time point for measurement is after 30 min when the hemodynamics becomes relatively stable. This rationale aligns with the clinical practice of performing one hour or longer NMP to evaluate kidney transplantability [48]. The discrimination was poorest by measuring *sO*_*2*_ within a deeper cortical layer. Measurements at greater depths can be influenced by low signal-to-noise ratio and fluence coloring, which can affect the accuracy of the *sO*_*2*_ measurement [49].

The pathology results demonstrated that renal injury becomes more prominent during NMP which is also confirmed by the literature [50]. Unlike the histological and biochemical analysis, PA can provide information about the kidney viability already after 30 min of NMP which is more practical than waiting for the laboratory results and prevents deterioration of kidney quality during prolonged NMP.

The experimental results demonstrate the correlation between *sO*_*2*_ and viability parameters such as RBF, *VO*_*2*_, and *CrCl* of the kidneys in Group 2. This finding indicates *sO*_*2*_ measured by PA imaging reflects the inner physiology of a kidney. The level of *CrCl* in Group 2 does not correlate with *sO*_*2*_. This further supports our conclusion that *sO*_*2*_ can discriminate between non-functioning and functioning transplants.

The correlation of *sO*_*2*_ with RBF, along with the significant difference in RBF between Group 1 and Group 2 after 2 h of NMP, suggests a direct relation between those two metrics. Despite the correlation between the metrics, there are two principal differences: Firstly, unlike RBF, the *sO*_*2*_ metric provides information about the oxygenation of the kidney cortex. Meanwhile, RBF depends on the size of vasculature which is not linearly related to cortical perfusion. Secondly, unlike RBF, PA can locally quantify *sO*_*2*_ and it allows to identify regions of occluded vasculature which is supported by our previous analysis of laser speckle dynamics in kidneys during NMP [34].

The proposed approach can be compared with alternative recently proposed optical-based imaging modalities: LSCI, fluorescence angiography, and hyperspectral imaging. In contrast with the abovementioned modalities, PA can quantify *sO*_*2*_ at up to a few centimeters’ depth whilst the optical imaging modalities are limited by a few millimeters. Besides, unlike PA, fluorescent angiography only measures perfusion within the cortical area without quantification of oxygen saturation. Notwithstanding, provided that the cortical perfusion corresponds to the *sO*_*2*_ level and the measurement is made only at 2 mm depth, we assume that those modalities can be equally used for the kidney quality assessment.

The macroscopic appearance of all kidneys during NMP was normal without showing perfusion insufficiency. Evaluation of the macroscopic appearance is a subjective method for evaluation of perfusion in the superficial layer of the cortex as we also demonstrated in [34]. The difference in blood volume reaching the cortical area in the kidneys from Group 1 and Group 2 is insufficient to make the discrimination only with the naked eye. Besides, low-oxygenated and highly oxygenated blood are also hard to distinguish.

Similarly, the histological examination of biopsies did not reveal significant differences between the two groups, despite some kidneys from Group 1 being graded as 3 after NMP. Two potential factors may contribute to this grading discrepancy. Firstly, individuals vary in their tolerance to warm ischemia, and this divergence in response could account for unexpected pathology grading. Secondly, renal biopsies are prone to sampling variability. Given that biopsy reflects only a small portion of the kidney, it may not comprehensively depict the overall condition, leading to discrepancies in pathology grading.

Currently, renal biopsy is still the gold standard for accurate pre-transplant quality assessment. However, it is important to note that besides its limitation of being unable to characterize the entire kidney and its invasive nature, renal biopsy may not be as sensitive as PA imaging since it cannot assess renal function, and has been documented to overestimate kidney damage [16]. Therefore, while renal biopsy continues to hold value in certain aspects, PA imaging provides a more refined approach to assess renal function during NMP.

Our study has several limitations to be addressed. Firstly, the use of slaughterhouse kidneys might result in higher interindividual variability in kidney viability and extent of renal injury, which could have interfered with our outcome measurements. Secondly, while porcine kidneys are commonly utilized in preclinical machine perfusion experiments, their anatomical and physiological characteristics differ from human kidneys. It is important to exercise caution when extrapolating our findings to clinical scenarios. Moreover, due to the sources of porcine kidneys, we were unable to perform transplantation and determine the definitive outcomes of transplant function. Further research should focus on applying PA imaging techniques to experimental kidneys intended for transplantation to validate our findings.

## 5 Conclusion

It is feasible to apply photoacoustic imaging on kidney viability assessment during normothermic machine perfusion. Low cortical oxygenation can identify kidneys with a high risk of inferior function and transplant failure. The presented technique has a high potential for clinical use since it can support organ selection in a transplantation setting.

## Funding

The work was supported by the “PICA HEART” project, funded by Health∼Holland (project number LSH-TKI EMC20024), and by the “HOST” project funded by Dutch Research Council (NWO, project number 20719).

## CRediT authorship contribution statement

**Anton V. Nikolaev:** Writing – review & editing, Writing – original draft, Methodology, Investigation, Formal analysis, Conceptualization. **Yitian Fang:** Writing – original draft, Investigation, Formal analysis. **Jeroen Essers:** Writing – review & editing, Project administration, Funding acquisition. **Kranthi M. Panth:** Investigation. **Gisela Ambagtsheer:** Investigation. **Marian C. Clahsen-van Groningen:** Investigation. **Robert C. Minnee:** Supervision, Methodology, Conceptualization. **Gijs van Soest:** Writing – review & editing, Supervision, Project administration, Methodology, Conceptualization. **Ron W.F. de Bruin:** Writing – review & editing, Supervision, Methodology.

## Declaration of Competing Interest

The authors declare that they have no known competing financial interests or personal relationships that could have appeared to influence the work reported in this paper.

## Data Availability

Data will be made available on request.

## References

[1] Tonelli M. (2011). Systematic review: kidney transplantation compared with dialysis in clinically relevant outcomes. Am. J. Transpl..

[2] *Organ Procurement & Transplantation Network*; Available from: 〈https://optn.transplant.hrsa.gov/〉.

[3] *Summary statistics on organ transplants, wait-lists and donors.* Canadian Institute for Health Information; Available from: 〈https://www.cihi.ca/〉.

[4] *Nederlandse Transplantatie Stichting*; Available from: 〈https://www.transplantatiestichting.nl/〉.

[5] *Eurotransplant: statistics report library*. Available from: statistics.eurotransplant.org.

[6] N.H.S. Blood and Trasnplant, *AnnualActivity Report*; Available from: 〈https://www.odt.nhs.uk/〉.

[7] Scandiatransplant; Available from: 〈http://www.scandiatransplant.org/〉.

[8] Lee S. (2019). Factors affecting mortality during the waiting time for kidney transplantation: a nationwide population-based cohort study using the Korean Network for Organ Sharing (KONOS) database. PLoS One.

[9] Port F.K. (2002). Donor characteristics associated with reduced graft survival: an approach to expanding the pool of kidney donors. Transplantation.

[10] Lomero M. (2020). Donation after circulatory death today: an updated overview of the European landscape. Transpl. Int.

[11] Hamed M.O. (2015). Early graft loss after kidney transplantation: risk factors and consequences. Am. J. Transpl..

[12] Legendre C., Canaud G., Martinez F. (2014). Factors influencing long-term outcome after kidney transplantation. Transpl. Int.

[13] Foley M.E. (2023). The impact of combined warm and cold ischemia time on post-transplant outcomes. Can. J. Kidney Health Dis..

[14] Girolami I. (2020). Pre-implantation kidney biopsy: value of the expertise in determining histological score and comparison with the whole organ on a series of discarded kidneys. J. Nephrol..

[15] von Moos S. (2020). Assessment of organ quality in kidney transplantation by molecular analysis and why it may not have been achieved. Yet. Front. Immunol..

[16] Aubert O. (2019). Disparities in acceptance of deceased donor kidneys between the United States and France and estimated effects of increased US acceptance. JAMA Intern Med.

[17] Hosgood S.A. (2023). Normothermic machine perfusion versus static cold storage in donation after circulatory death kidney transplantation: a randomized controlled trial. Nat. Med..

[18] Sjekavica I. (2018). Radiological imaging in renal transplantation. Acta Clin. Croat..

[19] Viazzi F. (2014). Ultrasound Doppler renal resistive index: a useful tool for the management of the hypertensive patient. J. Hypertens..

[20] Vernuccio F. (2018). CT evaluation of the renal donor and recipient. Abdom. Radio..

[21] Hohenwalter M.D. (2001). Renal transplant evaluation with MR angiography and MR imaging. Radiographics.

[22] Gerken A.L.H. (2022). Quantitative assessment of intraoperative laser fluorescence angiography with indocyanine green predicts early graft function after kidney transplantation. Ann. Surg..

[23] Rother U. (2017). Dosing of indocyanine green for intraoperative laser fluorescence angiography in kidney transplantation. Microcirculation.

[24] Hysi E. (2020). Photoacoustic imaging of kidney fibrosis for assessing pretransplant organ quality. JCI Insight.

[25] Zhou Y., Yao J., Wang L.V. (2016). Tutorial on photoacoustic tomography. J. Biomed. Opt..

[26] Riksen J.J.M., Nikolaev A.V., van Soest G. (2023). Photoacoustic imaging on its way toward clinical utility: a tutorial review focusing on practical application in medicine. J. Biomed. Opt..

[27] Mazilescu L.I. (2022). Normothermic ex vivo kidney perfusion for human kidney transplantation: first North American results. Transplantation.

[28] Hamelink T.L. (2022). Renal Normothermic Machine Perfusion: The Road Toward Clinical Implementation of a Promising Pretransplant Organ Assessment Tool. Transplantation.

[29] Hosgood S.A., Brown R.J., Nicholson M.L. (2021). Advances in kidney preservation techniques and their application in clinical practice. Transplantation.

[30] Pool M.B.F. (2021). Prolonged ex-vivo normothermic kidney perfusion: the impact of perfusate composition. PLoS One.

[31] Markgraf W. (2018). Algorithms for mapping kidney tissue oxygenation during normothermic machine perfusion using hyperspectral imaging. Biomed. Tech. (Berl..

[32] Markgraf W. (2020). Algorithm for mapping kidney tissue water content during normothermic machine perfusion using hyperspectral imaging. Algorithms.

[33] Schutter R. (2021). Magnetic resonance imaging assessment of renal flow distribution patterns during ex vivo normothermic machine perfusion in porcine and human kidneys. Transpl. Int.

[34] Fang, Y., et al., *Real-time laser speckle contrast imaging measurement during normothermic machine perfusion in pretransplant kidney assessment.* Lasers in Surgery and Medicine. **n/a**(n/a). 10.1002/lsm.23715 37555246

[35] Venema L.H. (2022). Impact of Red Blood Cells on Function and Metabolism of Porcine Deceased Donor Kidneys During Normothermic Machine Perfusion. Transplantation.

[36] Lohmann S. (2019). A pilot study of postoperative animal welfare as a guidance tool in the development of a kidney autotransplantation model with extended warm ischemia. Transpl. Direct.

[37] Lignell S. (2021). Improved normothermic machine perfusion after short oxygenated hypothermic machine perfusion of ischemically injured porcine kidneys. Transpl. Direct.

[38] Abdulkader R.C., Liborio A.B., Malheiros D.M. (2008). Histological features of acute tubular necrosis in native kidneys and long-term renal function. Ren. Fail.

[39] Urbanellis P. (2020). Significant dysfunction of kidney grafts exposed to prolonged warm ischemia is minimized through normothermic ex vivo kidney perfusion. Transpl. Direct.

[40] Markgraf W., Malberg H. (2022). Preoperative function assessment of ex vivo kidneys with supervised machine learning based on blood and urine markers measured during normothermic machine perfusion. Biomedicines.

[41] Ogurlu B. (2023). Prolonged controlled oxygenated rewarming improves immediate tubular function and energetic recovery of porcine kidneys during normothermic machine perfusion. Transplantation.

[42] Nicholson M.L., Hosgood S.A. (2013). Renal transplantation after ex vivo normothermic perfusion: the first clinical study. Am. J. Transpl..

[43] Jochmans I. (2011). The prognostic value of renal resistance during hypothermic machine perfusion of deceased donor kidneys. Am. J. Transpl..

[44] Ban S. (2018). Optical properties of acute kidney injury measured by quantitative phase imaging. Biomed. Opt. Express.

[45] Li W. (2019). in *Optical Coherence Imaging Techniques and Imaging in Scattering Media III*.

[46] McGill R., Tukey J.W., Larsen W.A. (1978). Variations of box plots. Am. Stat..

[47] Yin M. (2002). Different patterns of renal cell killing after warm and cold ischemia. Ren. Fail.

[48] Hosgood S.A. (2018). Normothermic machine perfusion for the assessment and transplantation of declined human kidneys from donation after circulatory death donors. Br. J. Surg..

[49] Hochuli R. (2019). Estimating blood oxygenation from photoacoustic images: can a simple linear spectroscopic inversion ever work?. J. Biomed. Opt..

[50] Huijink T.M. (2023). Loss of endothelial glycocalyx during normothermic machine perfusion of porcine kidneys irrespective of pressure and hematocrit. Transpl. Direct.

