# Supplementary material for: Pre-transplant kidney quality evaluation using photoacoustic imaging during normothermic machine perfusion
Source: Photoacoustics. 2024 Feb 9;36:100596. doi: 10.1016/j.pacs.2024.100596 (PMC10877941; doi:10.1016/j.pacs.2024.100596)
Supplement: Supplementary file 1 — Supplementary material [file mmc1.docx]

# Appendix

*Table S1. Components of the normothermic machine perfusion perfusate*

| **Priming** | **Volume (mL)** |
| --- | --- |
| Ringer’s lactate (Baxter, Utrecht, the Netherlands) | 300 |
| Autologous leukocyte-depleted blood | 500 |
| **Additives** |  |
| 200 g/L albumin (Prothya Biosolutions B.V., the Netherlands) | 20 |
| 8.4% sodium bicarbonate (B. Braun Melsungen AG, Germany) | 10 |
| 5% glucose (Baxter, Utrecht, the Netherlands) | 10 |
| Verapamil (Sigma-Aldrich, Zwijndrecht, the Netherlands) | 1 |
| Dexamethasone (Centrafarm, Etten-Leur, the Netherlands) | 0.3 |
| 90mg creatinine (Sigma-Aldrich, Zwijndrecht, the Netherlands) | - |
| **Infusion 20mL/h** |  |
| Aminoplasmal (B. Braun Melsungen AG, Germany) | 45 |
| 8.4% sodium bicarbonate (B. Braun Melsungen AG, Germany) | 1.5 |
| 100 U/mL insulin (NovoRapid^®^, Bagsværd, Denmark) | 0.5 |

Table S2. Histopathological grading for each kidney with correspondent sO_2_.

| Sample # | Group# | WIT (min) | Mean sO2 (%) | | | Histology grade | |
| --- | --- | --- | --- | --- | --- | --- | --- |
|  |  |  | 30 min | 60 min | 110 min | Before NMP | After NMP |
| 1 | 1 | 30 | 20 | 26 | 29 | 2 | 2 |
| 2 | 1 | 75 | 17 | 21 | 20 | 2 | 2 |
| 3 | 1 | 75 | 18 | 28 | 28 | 2 | 2 |
| 4 | 1 | 30 | 19 | 27 | 31 | 2 | 3 |
| 5 | 1 | 30 | 27 | 45 | 56 | 2 | 2 |
| 6 | 1 | 30 | 15 | 22 | 22 | 2 | 3 |
| 7 | 1 | 75 | 16 | 18 | 21 | 3 | 3 |
| 8 | 1 | 75 | 21 | 47 | 59 | 2 | 2 |
| 9 | 1 | 30 | 43 | 57 | 65 | 2 | 2 |
| 10 | 1 | 75 | 21 | 46 | 45 | 2 | 2 |
| 11 | 1 | 30 | 19 | 44 | 61 | 2 | 2 |
| 12 | 1 | 30 | 30 | 62 | 72 | 2 | 3 |
| 13 | 1 | 75 | 19 | 45 | 49 | 2 | 2 |
| 14 | 1 | 30 | 23 | 29 | 30 | 2 | 2 |
| 15 | 1 | 75 | 21 | 24 | 30 | 2 | 3 |
| 16 | 2 | 30 | 20 | 40 | 46 | 2 | 3 |
| 17 | 2 | 30 | 21 | 73 | 77 | 2 | 2 |
| 18 | 2 | 30 | 28 | 74 | 75 | 2 | 2 |
| 19 | 2 | 30 | 30 | 54 | 63 | 2 | 2 |
| 20 | 2 | 75 | 27 | 68 | 67 | 2 | 2 |
| 21 | 2 | 30 | 52 | 60 | 67 | 2 | 2 |
| 22 | 2 | 30 | 17 | 32 | 39 | 2 | 3 |
